# Death of Mr. Samuel Cooper

**Published:** 1849-03

**Authors:** 


### DEATH OF MR. SAMUEL COOPER.

Mr. Samuel Cooper, author of the "Surgical Dictionary," and "First Lines of Surgery," died in London on the 9th of last December, in the 68th year of his age, by a singular coincidence, on the anniversary of the death of his friend and colleague, Mr. Liston. At an early age, Mr. Cooper received an appointment in the medical staff of the army, and was present in the memorable Walcheren expedition. At the time of his death he was Professor of Surgery in the University College of London, which chair he had filled for seventeen years. He was also surgeon and consulting surgeon to the University Hospital from the time of its foundation until he left it, in April last, in consequence of a misunderstanding with some of his colleagues, to the irritation and mortification resulting from which the attack of retrocedent gout which caused his death, is generally attributed in his own country.

